# Assisted reproductive technologies (ART) in water buffaloes

**DOI:** 10.21451/1984-3143-AR2018-0043

**Published:** 2018-08-03

**Authors:** Pietro S. Baruselli, Julia G. Soares, Bernardo M. Bayeux, Júlio C.B. Silva, Rodolfo D. Mingoti, Nelcio A.T. Carvalho

**Affiliations:** 1 Departamento de Reprodução Animal, Universidade de São Paulo, São Paulo, SP, Brazil; 2 Centro de Pesquisa em Urologia, Escola Paulista de Medicina, Universidade Federal de São Paulo, São Paulo, SP, Brazil.; 3 Unidade de Pesquisa e Desenvolvimento de Registro, Centro de Zootecnia Diversificada, Instituto de Zootecnia, Registro, SP, Brazil

**Keywords:** artificial insemination, embryo transfer, synchronization.

## Abstract

Our expanding knowledge of ovarian function during the buffalo estrous cycle has given new approaches for the precise synchronization of follicular development and ovulation to apply consistently assisted reproductive technologies (ART). Recent synchronization protocols are designed to control both luteal and follicular function and permit fixed-time AI with high pregnancy rates during the breeding (autumn-winter) and nonbreeding (spring- summer) seasons. Additionally, allow the initiation of superstimulatory treatments at a self-appointed time and provide opportunities to do fixed-time AI in donors and fixed-time embryo transfer in recipients. However, due the scarce results of *in vivo* embryo recovery in superovulated buffaloes, the association of ovum pick-up (OPU) with *in vitro* embryo production (IVEP) represents an alternative method of exploiting the genetics of high yeld buffaloes. Nevertheless, several factors appear to be critical to OPU/IVEP efficiency, including antral follicle population, follicular diameter, environment, farm and category of donor. This review discusses a number of key points related to the manipulation of ovarian follicular growth to improve assisted reproductive technologies in buffalo.

## Introduction

The combined use of assisted reproductive technologies (ART), such as, timed-artificial insemination (TAI), superstimulation (SOV), ovum pick-up (OPU), *in vitro* embryo production (IVEP) and timed-embryo transfer (TET) has a great potential to improve reproductive outcomes and disseminate selected genetics, improving milk and beef production in buffalo herds.

However, the success of ART is closely related to the control of ovarian follicular development and ovulation. Buffalo is a seasonal reproductive species and becomes sexually active in response to a decreasing day length (short-days) in late summer to early autumn (Zicarelli, 1997). During the nonbreeding season, buffalo often exhibit anestrus, which extends the anovulatory period and consequently, reduces reproductive performance (Zicarelli, 2007).

In recent decades, several therapies have been proposed for manipulating ovarian follicle growth and ovulation in buffalo, regardless of reproductive seasonality (Baruselli *et al*., 2007; Campanile *et al*., 2010; Carvalho *et al*., 2016). These hormonal manipulations have been successfully used to optimize the reproductive outcomes following the application of various biotechnologies.

This review aims to elucidate some factors that affect the efficiency of assisted reproductive technologies (ART) in buffalo.

## Ovarian physiology in buffalo

**Figure 1:**
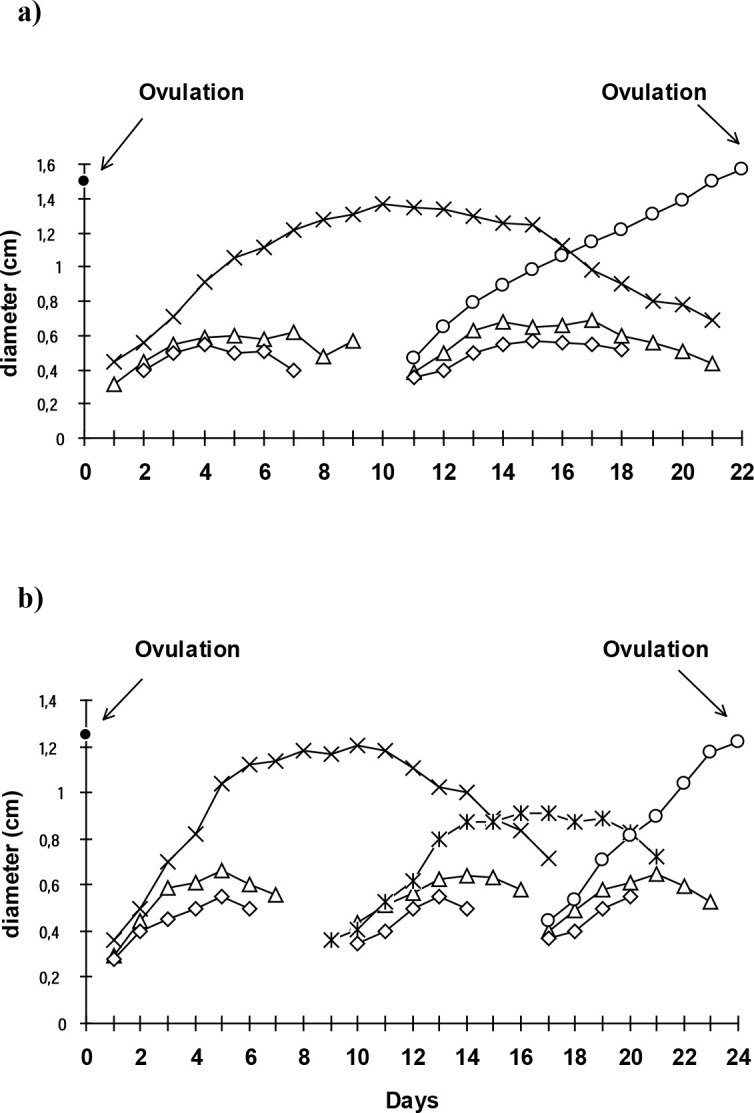
Standardized diameters of ovarian follicles (dominant follicle, largest and second largest subordinate follicle) in buffalo cows with a) two wave (n = 19) and b) three wave (n = 10) estrous cycles. Adapted from Baruselli *et al*. (1997).

The understanding of follicular dynamics in buffalo is necessary for developing new techniques and improving the currently used regimens for the manipulation of the estrous cycle. Ovarian follicular dynamics in buffalo are similar to those in cattle. The 2- wave cycle is the most common in buffalo (63.3%; Fig. 1; Baruselli *et al*., 1997) and the follicle deviation occurs 2.6 days after ovulation, when the diameters of the dominant and subordinate follicle are 7.2 and 6.4 mm, respectively (Gimenes *et al*., 2011). As in cattle, the number of waves in a cycle is also associated with the luteal phase and with the estrous cycle length.

However, the number of follicles recruited per follicular wave is lower in buffalo than in cattle (Baruselli *et al*., 1997; Gimenes *et al*., 2009; Campanile *et al*., 2010). The number of primordial cells in buffalo ovaries varies from 10,000 to 12,000 (Danell, 1987), which is about 10-fold lower than in cattle (Manik *et al*., 2002). Furthermore, it was verified that 92 to 95% of follicles are estrogen inactive/atretic at random stages of the reproductive cycle. Van Ty *et al*. (1989) also observed the existence of a lesser number of antral follicles in buffalo, when compared to cattle. These authors found that buffalo ovaries have about 20% of the number of antral follicles found in cattle (47.5 ± 23.8 *vs*. 233.0 ± 95.8; P < 0.002).

## Pharmacological control of follicular development and ovulation

### Prostaglandin F2α (PGF)

Estrus synchronization with prostaglandin F2α (PGF2α) is an effective and economical tool for induction of luteal regression, improving the estrous detection efficiency and the use of ART in buffaloes. Studies have shown that PGF2α treatment caused 100% luteolysis in buffalo (plasma progesterone <1 ng/ml within 48 h of administration), regardless of the luteal phase (early or late luteal phase; 6-9 or 11-14 days after estrus, respectively, (Porto Filho *et al*., 2014). Ovulation can occur up to 6 days after PGF2α administration, depending on the responsiveness of the corpus luteum (CL) and the stage of ovarian follicle development at the time of PGF2α treatment (Porto Filho *et al*., 2014). However, the major limitation of PGF2α in buffalo to apply efficient ART is the poor estrous behavior, and the lack of efficiency in females without a responsive CL (e.g., females within 5-6 days of a previous estrus) or in pre-pubertal heifers and postpartum anestrous cows. These particularities compromise the efficient use of only PGF2α treatment in reproductive programs in buffaloes.

### GnRH

The GnRH administration induces the emergence of a new follicular wave after induction of ovulation in cattle (Macmillan and Thatcher, 1991; Twagiramungu *et al*., 1992a, b,^1995^; Wolfenson *et al*., 1994; Schmitt *et al*., 1996). This information became the basis for subsequent development of programs to control timed ovulation.

In buffalo, 60.6% (20/33) of the cows ovulated after GnRH treatment at random stages of estrous cycle (Baruselli *et al*., 2013). The responses of GnRH depend on the diameter of the largest follicle at the moment of the treatment (Neglia *et al*., 2016). Buffalo that ovulated after GnRH treatment presented a larger follicle than animals that did not ovulate (9.5 ± 1.7 *vs*. 6.7 ± 2.4 mm; P < 0.01). However, no effect of the progesterone (P4) concentrations at the time of GnRH treatment and the GnRH dose (10 *vs*. 20 μg of buserelin) on the ovulation rate and the time of ovulation were observed. Furthermore, the interval between GnRH treatment and ovulation was between 28 to 33.0 h (Berber *et al*., 2002; Baruselli *et al*., 2003b; Campanile *et al*., 2008; Jacomini *et al*., 2014), similar to the interval observed in cattle (Wiltbank and Pursley, 2014).

### Estradiol plus progesterone to synchronize wave emergence

The combination of progesterone (P4) and estradiol (E2) treatment induces follicular atresia by suppressing FSH and LH release after the treatment and then synchronous emergence of a new follicular wave in response to the subsequent FSH release in cattle (reviewed by Bó *et al*., 2003) and buffaloes (reviewed in Baruselli *et al*., 2007) was observed.

The administration of 1 mg (Bartolomeu, 2003) or 1, 2.5 or 5.0 mg of estradiol benzoate (Moura, 2003) in progestin-treated buffalo results in emergence of a new follicular wave between 3 to 6 days after treatment in more than 90% of buffalo cows. However, a delay in the onset of follicular wave (8.7 ± 0.27 days) was observed when estradiol valerate was administrated (Bartolomeu, 2003). Treatment with P4 + E2 can be used efficiently to synchronize the emergence of a new follicular wave in buffaloes.

### Equine chorionic gonadotropin (eCG)

The treatment with equine chorionic gonadotropin (eCG) has been demonstrated as an alternative to increase final follicular development (follicular growth from luteolysis to ovulation) and pregnancy per TAI, mostly in anestrous buffalo during the non-breeding season (Carvalho *et al*., 2013). In buffalo with insufficient pulsatile release of LH to support the final stages of ovarian follicular development, treatment with eCG can improve the ovulatory response to the synchronization protocol and pregnancy outcome. The use of eCG in the synchronization protocol increases the diameter of the dominant follicle at TAI (13.7 ± 0.4 *vs*. 12.6 ± 0.6 mm, P = 0.09) and the ovulation rate (66.7 *vs*. 44.8%; P = 0.05). Moreover, eCG treatment results in increased CL diameter (15.8 ± 0.92 *vs*. 12.7 ± 0.77 mm, P = 0.03), increased P4 concentrations (0.59 ± 0.08 *vs*. 0.27 ± 0.05 ng/ml, P = 0.01) at the subsequent diestrus and increased pregnancy rate (52.7 *vs*. 39.4%, P = 0.03; Carvalho *et al*., 2013).

### LH, hCG, GnRH and estradiol benzoate to synchronize ovulation

After luteolysis, synchronization protocols require the use of inducers of ovulation to achieve a synchronized ovulation. Timed artificial insemination (TAI) protocols generally incorporate gonadotropin releasing hormone (GnRH), luteinizing hormone (LH), human chorionic gonadotropin (hCG) and estradiol esters to synchronize ovulation. The endocrine and follicular responses in buffalo to these different treatments are presented in Table 1.

All treatments for ovulation induction have satisfactory results in buffalo, with only particularities in the endocrine and follicular responses. Plasma P4 concentration at the subsequent diestrus was lower in GnRH (2.94 ± 1.51 ng/ml) than in hCG (4.02 ± 2.34 ng/ml; P < 0.05) treated buffalo for ovulation induction (Carvalho *et al*., 2007b). Furthermore, there is evidence that EB induces a greater release of LH compared with GnRH (Berber *et al*., 2007). and pre-exposure to P4 before EB administration anticipated the preovulatory- like LH surge in buffalo cows (Jacomini *et al*., 2014).

**Table 1:** Interval between treatment to induce ovulation and peak of LH, time to ovulation and ovulation rate in buffalo.

Treatment	Interval treatment to LH surge (h)	Interval treatment to ovulation (h)	Ovulation rate (%)	Reference
LH	-	24	93	Berber *et al*., 2002
hCG	-	24	81	Baruselli *et al*., 2003a; Carvalho *et al*., 2007a, b
GnRH	1-3	26-28	75-85	Berber *et al*., 2002, 2007; Baruselli *et al*., 2003b; Carvalho *et al*., 2013, 2017; Jacomini *et al*., 2014
Estradiol Benzoate	23-27	44	78-82	Berber *et al*., 2007; Jacomini *et al*., 2014; Carvalho *et al*., 2017

## ART for artificial insemination

Artificial insemination (AI) has proven to be a reliable technology for buffalo producers to improve genetic progress and control venereal diseases in their herds. However, the traditional AI program is impaired by the low estrous detection efficiency due to the poor manifestation of the symptoms of estrus in buffalo and to operational difficulties to detect estrus (Baruselli *et al*., 2007). Currently, timed artificial insemination (TAI) can be applied routinely in the reproductive programs on farms. TAI protocols are designed to control of both luteal and follicular function, permitting the TAI without estrus detection with satisfactory pregnancy per AI (P/AI), during the breeding and non-breeding season.

Among the hormonal therapies developed for cattle, GnRH plus PGF2a-based TAI protocols (Ovsynch; Pursley *et al*., 1995) resulted in follicular response with effective synchronization of ovulation in cycling buffaloes during the breeding season (Baruselli *et al*., 2003b). However, when the Ovsynch protocol was used in anestrous buffaloes (without CL), results were inferior to those obtained with cycling buffaloes. Souza *et al*. (2015b) verified that buffaloes without a CL at the beginning of the Ovsynch protocol responded poorly to the first (42.0 *vs*. 89.8% ovulation rate) and second (52.0 *vs*. 87.8% ovulation rate) GnRH treatments, and this resulted in a lower pregnancy rate after TAI (20.0 *vs*. 65.3%, respectively) compared to the animals with a CL. Results of several other studies revealed a high incidence of anestrus during the nonbreeding season (spring and summer), and lower pregnancy rates after TAI were reported when the Ovsynch protocol was used (7.0-30.0%; Baruselli *et al*., 1999, 2002, 2003b, 2007; De Rensis *et al*., 2005; Ali and Fahmy, 2007; Vecchio *et al*., 2013). Therefore, during the nonbreeding season, when a high incidence of anestrus is expected, lower pregnancy rates are encoutered in buffaloes synchronized with the Ovsynch protocol for TAI. On the contrary, studies have demonstrated similar pregnancy per TAI in both breeding and nonbreeding seasons after the use P4, E2, and eCG- based protocols (Baruselli *et al*., 2013; Carvalho *et al*., 2013; Monteiro, 2018; Table 2).

These data demonstrate that it is possible to establish an effective AI program in buffaloes throughout the year, however it is relevant to understand the interactions between ovulation synchronization treatments and the season of the year.

**Table 2:** Pregnancy per AI in lactating buffalo cows subjected to GnRH plus PGF2α (Ovsynch) or P4/E2 and eCG based protocol during the breeding and nonbreeding season.

	Breeding season	Nonbreeding season	P value
GnRH plus PGF2 (Ovsynch) ^1^			
Pregnancy rate per TAI	48.8% (472/967)	6.9% (6/86)	0.001
P4/E2 and eCG^2^			
Pregnancy rate per TAI	66.7% (112/168)	62.7% (111/177)	0.31

1 Baruselli *et al*. (2003b); Monteiro (2018).

## ART for embryo production

Reproductive technologies, such as superstimulation for *in vivo* embryo production and ovum-pick-up (OPU) for *in vitro* embryo production (IVEP) can rapidly enhance genetics in buffaloes through both the female and male superior lineage. The *in vivo-*derived (IVD) embryo production has been shown to be feasible in buffalo, however low efficiency and limited commercial application has been documented (Baruselli *et al*., 2000; Campanile *et al*., 2010). Currently, a series of recent studies have demonstrated the potential of *in vitro* embryo production (IVP) in buffalo. Studies on the particularities of these biotechnologies in buffalo will be discussed.

### Production of in vivo-derived (IVD) embryos

The multiple ovulation followed by TAI for *in vivo* embryo production is a technique that generates greater numbers of embryos per donor in cattle (Mapletoft *et al*., 2002). These techniques, which are associated with ET to recipients, are powerful tools to accelerate the gain in genetic programs (Bó *et al*., 2002; Baruselli *et al*., 2011). However, buffalo donors generally have lower embryo recovery rates than bovines. While buffaloes have shown follicular responses after superovulation treatment (mean of 15 follicles >8 mm), only a moderate ovulation rate (approximately 60%) and CL yield at the time of flushing (approximately 9 CL) and low embryo recovery rates (34.8%) have been obtained (Baruselli *et al*., 2000). The embryo recovery rate in superovulated buffaloes (approximately 20 to 40%) is lower than in cattle (63 to 80%; Boland *et al*., 1991; Adams, 1994; Vos *et al*., 1994; Shaw *et al*., 1995). This divergence in embryo recovery rates was hypothesized to be related to a failure of oocyte capture and/or of oocyte transport along the oviduct (Baruselli *et al*., 2000). In rabbits, the administration of sequential doses of PGF_2α_ during the periovulatory period stimulated the contraction of oviduct smooth muscles, allowing the activation of the oviduct fimbriae to capture the oocytes (Osada *et al*., 1999). Based on this observation, our research group (Soares, 2015) performed an experiment that evaluated the use of PGF2α (injectable or using a mini osmotic pump; OP) during the periovulatory period in superovulated buffaloes. However, no differences were found on the total number of recovered structures (G-CONT = 2.1 ± 0.8 *vs*. GPGF- IM = 2.1 ± 0.6 *vs*. G-PGF-OP = 1.4 ± 0.4; P = 0.58). The low embryo production per donor impairs the use of this biotechnology by buffalo producers.

### In vitro *embryo production (IVEP)*

Due the scarce results of *in vivo* embryo recovery in superovulated buffaloes, the association of OPU with IVEP represents an alternative method of exploiting and multiplying genetic for superior merit (Boni *et al*., 1996; Neglia *et al*., 2003; Sá Filho *et al*., 2009). Historically, OPU-IVEP in buffaloes produced lower outcomes (Gasparrini, 2002; Sá Filho *et al*., 2009; Gimenes *et al*., 2010) than in bovines (Lonergan and Fair, 2008; Pontes *et al*., 2011). However, a series of recent studies have demonstrated the commercial potential of these techniques in the buffalo specie (Baruselli *et al*., 2013).

Two main biological problems seem to be related to the low efficiency of the OPU-IVEP technique in buffaloes: 1) low number of follicles on the ovary that results in low oocyte recovery per OPU and; 2) poor oocyte quality retrieved (only 27.3 to 31.3 % of oocytes are classified as viable (Campanile *et al*., 2003).

The first limitation can be related to the lower number of follicles recruited per follicular wave (Baruselli *et al*., 1997), as observed in studies comparing buffaloes with *Bos indicus* cattle (Ohashi *et al*., 1998; Gimenes *et al*., 2015). Additionally, a higher level of follicular atresia was reported (Danell, 1987; Van Ty *et al*., 1989) and, consequently, a lower number of total recoverable and viable oocytes. Buffaloes and cattle raised with contemporary nutrition and management were compared *post mortem* by Ohashi *et al*. (1998), and *in vivo* by Gimenes *et al*. (2015). In both studies, lower number of follicles and viable oocytes were observed in buffaloes than in *Bos indicus* cattle.

The second limitation can be attributed to a more fragile zona pellucida (Mondadori *et al*., 2010) and a more fragile bonding between cumulus cells and the oocyte (Ohashi *et al*., 1998; Gasparrini, 2002) in buffaloes than in cattle.

Thus, to improve oocyte quality and recovery, studies were conducted by our research group to upgrade this biotechnology in buffaloes. Initially, we tested the hypothesis that bST could elevate circulating IGF-1 levels, promoting recruitment of a greater number of follicles and enhancing oocyte quality (Sá Filho *et al*., 2009). Although bST treatment resulted in greater numbers of aspirated follicles and retrieved oocytes per donor per session, reduced blastocyst production rate was observed (Ferraz *et al*., 2007, 2015; Sá Filho *et al*., 2009).

The phase of the estrous cycle is an important factor that directly influences the quantity and quality of oocytes obtained by OPU and, consequently IVP efficiency (Vassena *et al*., 2003). Thus, in another study buffalo (*Bubalus bubalis*), Nelore (*Bos indicus*) and Holstein (*Bos taurus*) heifers were synchronized to be submitted to OPU 1, 3 or 5 days after wave emergence. No effects were observed on the OPU- IVEP efficiency according to the different phases of the synchronized ovarian follicular wave in all genetic groups. However, the OPU-IVEP procedure was less efficient in buffalo and Holstein than in Nelore heifers (Gimenes *et al*., 2015).

The influence of season (winter; breeding season or summer; nonbreeding season) on oocyte viability (number of viable oocytes and mitochondrial DNA amount) was investigated in nulliparous and multiparous buffaloes (Macabelli *et al*., 2012). During summer, the amount of mtDNA was lower in oocytes from nulliparous than those from multiparous, but during winter mtDNA amount was greater in oocytes from nulliparous than those from multiparous. The mtDNA analyses do not suggest a negative effect of summer on oocyte viability in buffalo. Therefore, in tropical climates, the season would not appear to adversely affect oocyte quality and fertility. However, other studies carried out in buffalo showed an effect of season on either the number of follicles/viable oocytes or oocyte developmental competence, at different latitudes (Manjunatha *et al*., 2009; Di Francesco *et al*., 2011, 2012).

### Number of oocytes retrieved per buffalo and its relationship with in vitro embryo production and pregnancy

The number of antral follicles in the early follicular phase is directly correlated with the ovarian reserve (Frattarelli *et al*., 2000). Indeed, the antral follicular population (AFP) directly represents the follicle cohort in the ovaries, which is associated with the number of oocytes retrieved per OPU for IVEP.

A large variability of AFP is reported among different females, however AFP count is highly repeatable within animal (Burns *et al*., 2005; Ireland *et al*., 2007), and anti-Müllerian hormone (AMH) can be considered a reliable endocrine marker of ovarian reserve (Ireland *et al*., 2007, 2008; Monniaux *et al*., 2012). In cattle, circulating AMH concentration can help veterinarians to predict AFP in ovaries (Ireland *et al*., 2008; Rico *et al*., 2009; Batista *et al*., 2014), response to SOV treatments (Rico *et al*., 2009; Monniaux *et al*., 2010a, b; Souza *et al*., 2015a), and more recently as a marker to predict IVEP performance of *Bos taurus* (Guerreiro *et al*., 2014 Gamarra *et al*., 2015; Vernunft *et al*., 2015) and *Bos indicus* breeds (Guerreiro *et al*., 2014).

Aiming to determine the relation between AMH and AFP we recently conducted a study in buffalo and cattle (Baldrighi *et al*., 2014; Liang *et al*., 2016). Despite the high variability in AFP among individuals within each genetic group, the AFP count was greater in Gir (*Bos indicus*) than in Holstein (*Bos taurus*) and Murrah (*Bubalus bubalis*) heifers (P = 0.01). Similarly, AMH concentration was lower (P < 0.01) for Holstein and Murrah heifers than for Gir heifers. In spite of the differences between genetic groups, a positive relationship among AFP and AMH concentration was detected within buffalo. These studies suggest AMH as endocrine marker to predict AFP and IVEP performance in buffalo.

Recently we have studied the relationship between AFP and *in vitro* embryo production and pregnancy rate in buffalo. The number of oocytes recovered per OPU (analyzed by tertile) had no effect on viable oocyte and blastocyst rates (Table 3). However, the number of blastocysts per OPU was greater when higher number of oocytes were recovered per OPU. Pregnancy rate following ET in buffalo was lower in donors with greater amounts of oocytes retrieved per OPU.

**Figure 2:**
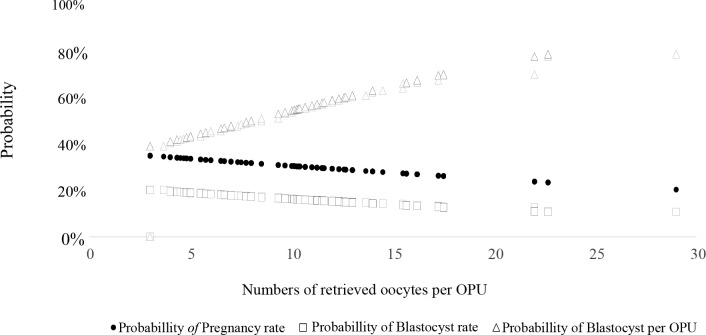
Probability of blastocyst rate (□), pregnancy rate (●) and blastocisty per OPU (∆) as a function of numbers of retrieved oocytes per OPU in Murrah buffalo (*Bubalus bubalis*) donors (n = 179). Probability_blastocyst_rate = EXP (-0.0375* Oocytes_retrived -1.2673) / [1+ EXP ( -0.0375 * Oocytes_retrived - 1.2673)]; P = 0.07; r2 = 0,02 Probability_pregnancy_rate = EXP (-0.0287 * Oocytes_retrived -0.5366) / [1+ EXP (-0.0287 * Oocytes_retrived - 0.5366)]; P = 0.41; r2 = 0.0025. Probability_blastocyst per OPU = EXP (+0.0891 * Oocytes_retrived -0.7164) / [1+ EXP (+0.0891 * Oocytes_retrived - 0.7164)]; P < 0.001; r2 = 0.35.

**Figure 3:**
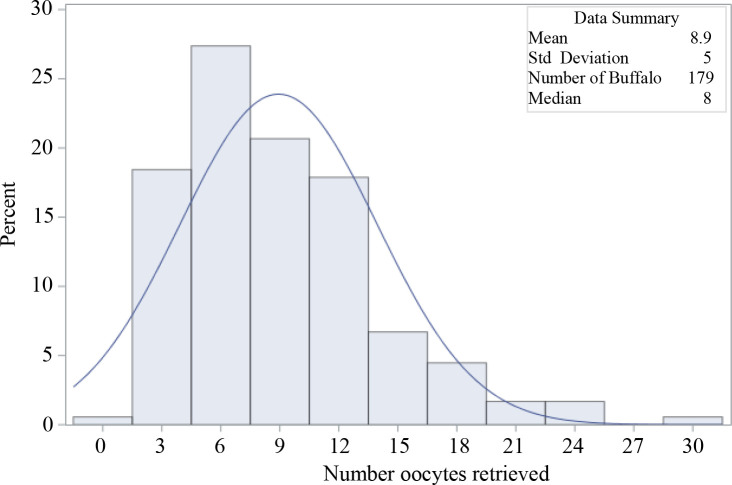
Distribution of oocytes retrieved per OPU in Murrah buffalo donor (n = 179).

The results demonstrate that the number of oocytes recovered per OPU had a minor effect after ET both on blastocyst rate and pregnancy rates. However as more oocytes are collected, the number of produced blastocysts improves (Fig. 2). These results highlight the relevance to identify donors with greater potential to oocyte recovery per OPU to assure greater IVEP success, especially in buffalo that yield fewer oocytes per OPU than bovine. There was great variation in the number of oocytes retrieved per OPU (from 0 to 30), with a mean of 8.9 ± 5.0 per donor (Fig. 3). Therefore, a holistic approach selecting donors with greater genetic value (through genomics) and oocyte population (through AMH assays or ultrasound for quantify AFP) is highly advisable.

**Table 3:** Effect of retrieved numbers of oocytes per OPU from Murrah buffalo (*Bubalus bubalis*) donors on IVEP.

Items		TERTILE		P value
	Low	Medium	High	
Tertile, n	60	59	60	-
Retrieved oocytes, n	4.1 ± 0.14^c^	8.2 ± 0.19^b^	14.5 ± 0.5^a^	<0.0001
Viable oocytes, n	2.1 ± 0.17^c^	3.9 ± 0.24^b^	7.7 ± 0.37^a^	0.0002
Viable oocyte rate, %	51.8	47.8	53.2	0.31
Blastocyst per OPU, n	0.83 ± 0.11^c^	1.19 ± 0.13^b^	2.17 ± 0.24^a^	<0.0001
Blastocyst rate, %^1^	20.3	14.5	14.9	0.15
Pregnancy rate, %	44.2 (22/50)^a^	29.6 (21/70)^ab^	25.3 (33/130)^b^	0.05

1 No. blastocysts/no. retrieved oocytes; Adapted from Soares *et al*. (2018); Centro de Pesquisa em Urologia, Escola Paulista de Medicina, São Paulo, SP, Brazil; unpublished data.

### Factors affecting OPU/IVF efficiency in buffaloes

Numerous factors may interfere with the efficiency of OPU/IVEP in buffaloes. Table 4 shows the effect of farm, category, postpartum period, reproductive status (pregnant or non-pregnant at the OPU) and BCS on IVEP production in buffalo donors (Carvalho *et al*., 2018; Unidade de Pesquisa e Desenvolvimento de Registro, Instituto de Zootecnia, Registro, SP, Brazil; unpublished data). The HPMIXED procedure of SAS through the best linear unbiased prediction (BLUP) analysis was utilized to rank sires, farms, category, postpartum period and BCS in terms of oocytes per OPU, number of blastocysts and blastocyst rate. Effects of farm (P = 0.05), category (P = 0.07) and reproductive status (P = 0.02) on the number of retrieved oocytes per OPU were found. Nulliparous and primiparous produced higher number of retrieved oocytes per OPU than multiparous. Furthermore, pregnant buffaloes (30 to 120 days of gestation) produced lower number of retrieved oocytes per OPU than non-pregnant. However, no effects were observed in the number of embryo produced per OPU and embryo rate (Table 4).

**Figure 4:**
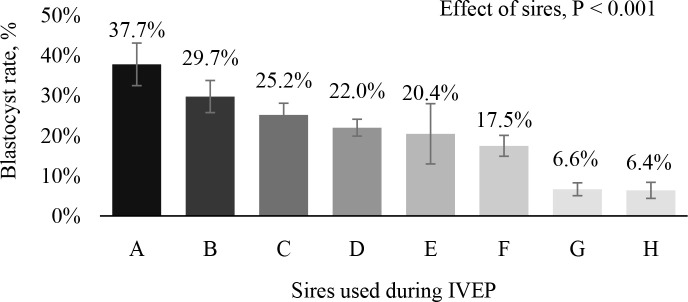
Blastocyst rate (%) according to sires used (n = 8) during IVEP from buffalo (*Bubalus bubalis*) donors (n = 379).

There is also a strong effect of the bull on the efficiency of IVF in buffaloes (Fig. 4). It is clear that semen used during *in vitro* procedures potentially influence IVEP and field fertility results (Watanabe *et al*., 2017). Top ranking sires yielded outstanding blastocyst rates, while poor sires produced low blastocyst rates.

**Table 4:** Effect of different variables in the IVEP production in buffalo donors.

Variable	Number of retrieved oocytes	P value	Embryo produced per OPU	P value	Embryo rate (%)	P value
Farm		0.05		0.75		0.54
A (n = 114)	9.6 ± 0.5^a^		1.7 ± 0.2		18.5%	
B (n = 269)	8.9 ± 0.3^ab^		1.7 ± 0.1		20.0%	
C (n = 38)	6.9 ± 0.9^b^		1.5 ± 0.3		26.4%	
Category		0.07		0.48		0.62
Nuliparous (n = 57)	10.2 ± 0.7		1.7 ± 0.2		17.9%	
Primiparous (n = 39)	11.1 ± 0.9		2.0 ± 0.3		21.2%	
Multiparous (n = 245)	8.34 ± 0.4		1.6 ± 0.1		18.4%	
Post partum period		0.92		0.45		0.26
≤117d (n = 68)	9.5 ± 0.8		2.1 ± 0.2		24.4%	
117d to 217d (n = 68)	9.1 ± 0.6		2.2 ± 0.3		17.7%	
>217d (n = 69)	8.5 ± 0.5		1.6 ± 0.2		26.0%	
Reproductive status		0.02		0.80		0.13
Pregnant (n = 52)	7.9 ± 0.6^b^		1.7 ± 0.2		23.3%	
Non pregnant (n = 139)	10.0 ± 0.5^a^		1.8 ± 0.1		17.5%	
BCS		0.98		0.88		0.44
≤3.0 (n = 25)	9.4 ± 1.3		2.0 ± 0.5		20.3%	
3.0 to 4.0 (n = 42)	9.6 ± 0.9		1.3 ± 0.3		16.8%	
>4.0 (n = 47)	9.8 ± 0.8		2.0 ± 0.3		19.1%	

### Superstimulation with FSH prior to ovum pick-up

Superstimulation with FSH previous to OPU has been used successfully for IVP programs in cattle, resulting in increased total embryo yields per OPU session (Goodhand *et al*., 1999; Sendag *et al*., 2008; Vieira *et al*., 2014), possibly because of the greater follicular diameters of the aspirated follicles. The FSH treatment for superstimulation can promote the growth of a homogeneous follicle population and to recover competent oocytes suitable for IVEP procedures.

**Figure 5:**
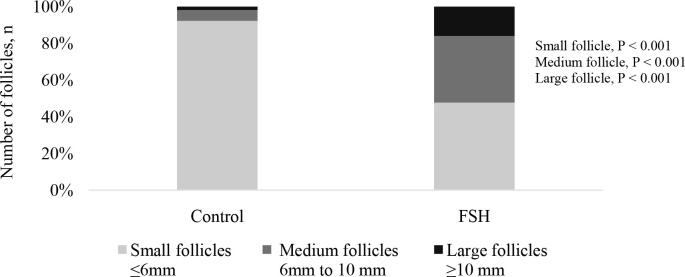
Proportion of small (<6 mm), medium (6-10 mm), and large follicles (>10 mm) in buffalo donor submitted to OPU with and without FSH superstimulation prior to OPU.

In buffalo, superstimulation with FSH prior to OPU increased the proportion of large and medium-sized follicles available for the OPU procedure (Fig. 5). Consequently, the treatment enhanced the proportion of oocytes suitable for culture and resulted in greater blastocyst rates and embryo yield per OPU-IVEP session (Table 5).

These results provide evidence that superstimulation with FSH increased the proportion of medium-sized follicles available for the OPU procedure. Consequently, the treatment also enhanced the proportion of viable oocytes for culture and resulted in greater blastocyst rates and embryo yield per OPU-IVP session in buffalo.

**Table 5:** Summary of oocyte and embryo production (mean ± SEM) after OPU-IVEP in control and p-FSH-treated buffalo donors (heifers, primiparous and multiparous).

Heifers			Primiparous		Multiparous			P value	
Item	Control	FSH	Control	FSH	Control	FSH	Treat	Cat	Treat^*^Cat
No.	18	18	15	15	21	21			
Total follicles aspirated, n	20.3 ± 2.4	18.3 ± 1.6	21.3 ± 4.4	17.7 ± 2.9	18.1 ± 2.2	17.6 ± 1.7	0.53	0.73	0.85
Total oocytes retrieved, n	11.7 ± 1.6	12.3 ± 1.0	11.5 ± 2.0	9.0 ± 1.2	8.7 ± 1.0	9.3 ± 1.2	0.85	0.05	0.46
Recovery rate, %	68%	73%	66%	55%	53%	53%	0.92	0.71	0.92
Viable oocyte, n	6.6 ± 1.3	7.8 ± 0.9	5.9 ± 1.5	5.67 ± 1.1	4.3 ± 0.7	5.6 ± 0.9	0.26	0.08	0.72
Viable rate, %	50%	58%	47%	56%	50%	57%	0.03	0.46	0.95
Embryo per OPU	1.8 ± 0.5	3.7 ± 0.7	2.4 ± 0.6	2.7 ± 0.8	2.0 ± 0.5	2.6 ± 0.7	0.07	0.25	0.22
Blastocyst rate, %	17%	34%	27%	28%	24%	32%	0.03	0.89	0.25

Adapted from Soares *et al.* (2018); Centro de Pesquisa em Urologia, Escola Paulista de Medicina, São Paulo, SP, Brazil; unpublished data.

### Buffalo calves as oocyte donors

With the advent of genomic technology in association with the traditional genetic evaluation, the use of calves as oocyte donors is an important strategy to accelerate genetic gain by decreasing generation intervals (Armstrong *et al*., 1992; Lohuis, 1995; Camargo *et al*., 2005). Several research groups have successfully produced viable embryos from prepubertal heifers (Armstrong *et al*., 1992; Revel *et al*., 1995; Fry *et al*., 1998; et al., 1998; Taneja *et al*., 2000; Baruselli *et al*., 2016) in cattle. However, there are some concerns that oocytes from young females have a lower developmental capacity than those from adult donors (Khatir *et al*., 1996; Presicce *et al*., 1997; Majerus *et al*., 1999; Palma *et al*., 2001). In buffalo, our group compared the embryo production of calves (from 2 to 4 months of age) in relation to prepubertal heifers (from 13 to 15 months of age) and lactating buffalo cows (Silva *et al*., 2017). The calves received sheep intravaginal P4 device (day 0) and were treated with 140 mg of FSH in 4 decreasing doses at 12h intervals on day 5 and day 6. Calves were aspirated on day 7 by laparoscopy (LOPU - Laparoscopy Ovum Pick Up) and prepubertal heifers and adult lactating cows by intravaginal follicular aspiration (OPU). Both LOPU and OPU were performed on the same day and the same sire was used for IVF. Data are shown in the Table 6.

The calves embryos produced (n = 8) were transferred to synchronized recipients at the São Paulo University Campus and three pregnancies were diagnosed (pregnant/transferred = 38%; 3/8) at the 30 and 60 days of gestation and three healthy calves were born, demonstrating the viability of this biotechnology for buffalo.

**Table 6:** Number oocytes retrieved and blastocysts produced (mean ± SEM) after LOPU-IVEP in buffalo donor calves and after OPU - IVEP in prepubertal heifers and cows.

	Category			P value
	Calves	Pre-pubertal heifers	Lactating cows	
No.	8	10	10	
Total oocytes retrieved, n	10.9 ± 3.3^ab^	15.5 ± 2.1^a^	5.8 ± 1.3^b^	0.007
Viable oocytes, n	7.6 ± 2.7	6.2 ± 1.6	3.2 ± 0.9	0.11
Viable oocytes rate, %^a^	63.9^a^	39.3^b^	54.1^a^	0.01
Total oocytes cleaved, n	2.7 ± 0,9	3.1 ± 0.7	2.1 ± 0.4	0.52
Cleavage rate, %^b^	30.3^ab^	20.8^b^	37.6^a^	0.04
Viable embryos, n	1.0 ± 0.6^b^	1.5 ± 0.3^a^	1.1 ± 0.4^ab^	0.02
Embryos rate, %^c^	5.1^b^	9.3^a^	15.4^a^	0.05

Adapted from Silva *et al*. (2017).

## Embryo recipient synchronization

The inefficiency in estrus detection, especially in buffalo, has limited its widespread application and greatly increased the cost of embryo transfer commercial operations. The incorporation of techniques designed to control follicular wave dynamics and ovulation reduces the problem of estrus detection and provides possibilities for the application of efficient FTET programs in buffalo. At unknown days of the estrous cycle (day 0), buffalo recipients were treated with intravaginal progesterone device plus 2 mg of EB (im). Nine days later (day 9), the P4 device was removed and the recipients received PGF and eCG (400 IU). On day 11, recipients were treated with GnRH and on day 17 recipients received a FTET (Saliba *et al*., 2013; Soares *et al*., 2015). The results showed similar efficiency for FTET when different categories of recipients (nulliparous, primiparous and multiparous) were used (Soares *et al*., 2015).

## Summary and conclusions

Currently there is technology overall to establish efficient programs for the use of ART in buffalo. The control of follicular wave emergence and ovulation at predetermined times, without estrus detection, has facilitated the AI programs and the donor and recipient management. Synchronization protocols are designed to control both luteal and follicular function and permit fixed-time AI with high pregnancy rates during the breeding (autumn-winter) and nonbreeding (spring and summer) seasons. The OPU/IVEP is showing promising results, and has become an alternative to superovulation for *in vivo* embryo production. The use of this biotechnology makes possible to promote a rapid enhancement in genetics through both the female and male lineage. Therefore, the ART are being established and can collaborate for genetic improvement and reproductive efficiency, increasing the meat and milk production of the buffalo herds.
